# Systematic review and subgroup analysis of the incidence of acute kidney injury (AKI) in patients with COVID-19

**DOI:** 10.1186/s12882-021-02244-x

**Published:** 2021-02-05

**Authors:** Zhenjian Xu, Ying Tang, Qiuyan Huang, Sha Fu, Xiaomei Li, Baojuan Lin, Anping Xu, Junzhe Chen

**Affiliations:** 1 Department of Nephrology, Sun Yat-sen Memorial Hospital of Sun Yat-sen University, Guangzhou, 510120 China; 2 Guangdong Provincial Key Laboratory of Malignant Tumor Epigenetics and Gene Regulation, Sun Yat-sen Memorial Hospital of Sun Yat-sen University, Guangzhou, 510120 China; 3 Department of Nephrology, The Third Affiliated Hospital, Southern Medical University, Guangzhou, 510630 China

## Abstract

**Background:**

Acute kidney injury (AKI) occurs among patients with coronavirus disease-19 (COVID-19) and has also been indicated to be associated with in-hospital mortality. Remdesivir has been authorized for the treatment of COVID-19. We conducted a systematic review to evaluate the incidence of AKI in hospitalized COVID-19 patients. The incidence of AKI in different subgroups was also investigated.

**Methods:**

A thorough search was performed to find relevant studies in PubMed, Web of Science, medRxiv and EMBASE from 1 Jan 2020 until 1 June 2020. The systematic review was performed using the meta package in R (4.0.1).

**Results:**

A total of 16,199 COVID-19 patients were included in our systematic review. The pooled estimated incidence of AKI in all hospitalized COVID-19 patients was 10.0% (95% CI: 7.0–12.0%). The pooled estimated proportion of COVID-19 patients who needed continuous renal replacement therapy (CRRT) was 4% (95% CI: 3–6%). According to our subgroup analysis, the incidence of AKI could be associated with age, disease severity and ethnicity. The incidence of AKI in hospitalized COVID-19 patients being treated with remdesivir was 7% (95% CI: 3–13%) in a total of 5 studies.

**Conclusion:**

We found that AKI was not rare in hospitalized COVID-19 patients. The incidence of AKI could be associated with age, disease severity and ethnicity. Remdesivir probably did not induce AKI in COVID-19 patients. Our systematic review provides evidence that AKI might be closely associated with SARS-CoV-2 infection, which should be investigated in future studies.

**Supplementary Information:**

The online version contains supplementary material available at 10.1186/s12882-021-02244-x.

## Background

Coronavirus disease 2019 (COVID-19), which is caused by severe acute respiratory syndrome coronavirus 2 (SARS-CoV-2), has led to more than 60 million infections and over 1 million deaths worldwide [1]. The mortality due to COVID-19 is particularly high among older patients with chronic diseases, including hypertension, diabetes, obesity, chronic kidney disease and cardiac disease [2]. In 2003, the incidence of acute kidney injury (AKI) in patients with SARS was reported to be 6.7, and 91.7% of patients who died were diagnosed with AKI as a complication [3]. Recent studies have suggested that the incidence of AKI during hospitalization in patients with COVID-19 has a wide range and that AKI is associated with a poor prognosis [4–6]. Continuous renal replacement therapy (CRRT) is usually required for critically ill COVID-19 patients, not only for the treatment of AKI but also to effectively eliminate the cytokine storm [7]. The need for CRRT in COVID-19 patients should be evaluated.

Given the current ongoing pandemic of COVID-19, there is a need to identify safe and effective treatment options. Remdesivir, a broad-spectrum antiviral agent, has been shown to have antiviral activity against several RNA viruses, including MERS-CoV and Ebola virus (EV) [8, 9]. As remdesivir was found to effectively inhibit SARS-CoV-2 in vitro and in a mouse model [10, 11], it has been authorized for the treatment of COVID-19 patients in some countries, including the United States [12]. The incidence of AKI in COVID-19 patients being treated with remdesivir is still uncertain. Overall, the exact incidence rate and characteristics of AKI associated with COVID-19 are not well understood. In this study, we performed a systematic review of the incidence of AKI in hospitalized patients with COVID-19.

## Methods

### Search strategy

A systematic literature search was performed using PubMed, Web of Science, medRxiv and EMBASE from 1 Jan 2020 until 1 June 2020 to summarize the incidence of AKI in patients hospitalized with COVID-19. Two authors independently carried out systematic literature searches employing the terms “kidney” OR “renal” OR “acute kidney injury” OR “acute renal failure” AND “COVID-19” OR “SARS-COV-2” to obtain the AKI incidence in patients hospitalized with COVID-19. No language restrictions were applied.

### Inclusion and exclusion criteria

Studies were included if they met the following criteria: 1) observational studies that reported the incidence of AKI in all hospitalized patients with COVID-19 and 2) observational studies or randomized, placebo-controlled trials (RCTs) that reported the incidence of AKI in hospitalized patients with COVID-19 being treated with remdesivir.

Studies that 1) were editorials, review articles or case reports, 2) were preprint articles, 3) had incomplete information about AKI, and 4) did not utilize the 2012 KDIGO criteria to define AKI were excluded.

### Quality assessment

The methodological quality of the retrospective cross-sectional studies was assessed independently by two reviewers (Chen and Xu) using the method of the Agency for Healthcare Research and Quality (AHRQ) (http://www.ncbi.nlm.nih.gov/books/NBK.

35,156). An item was scored as 0 if it was answered NO or UNCLEAR; if it was answered YES, then the item was scored as 1. Studies achieving a score of 8 or above were considered high quality. At the same time, the RCTs in our study were analysed using the Cochrane Collaboration tool (http://handbook-5-1.cochrane.org/). Studies were divided into groups A, B and C. Studies that were assigned to the A group were considered high quality.

### Statistical analysis

The systematic review was performed using the meta package in R (4.0.1). The incidence of AKI in COVID-19 patients (proportion) was used in our study. The incidences and their 95% CIs are presented as forest plots generated by the Metaprop function. Statistical heterogeneity among studies was assessed using the *I*^*2*^ statistic. The random-effects model was used if there was heterogeneity between studies (*I*^*2*^ < 50%); otherwise, the fixed-effects model was adopted. Rate consolidation was conducted using five methods (untransformed, log transformation, logit transformation, arcsine transformation, and Freeman-Tukey double arcsine transformation), and the logit transformation that yielded the results with the lowest I^2^ was selected for inclusion in our study. Sensitivity analysis was performed by the leave-one-out method. Peter’s test was performed to assess publication bias, and significance was determined by a *P* < 0.05.

## Results

### Literature search and study characteristics

**Fig. 1:**
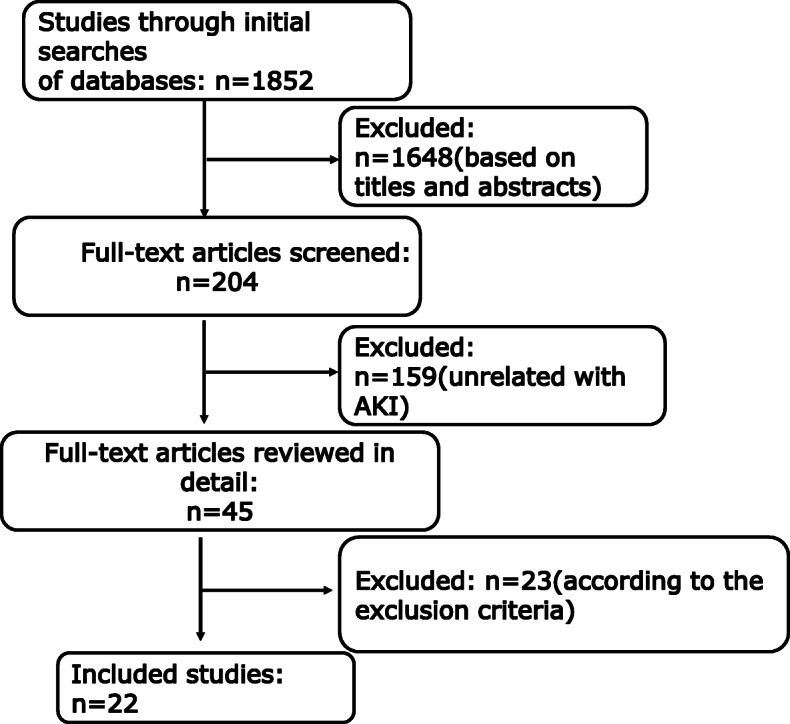
Flow diagram of studies identified, included, and excluded

A total of 1852 papers were identified according to our search criteria. After an initial round of exclusion based on titles and abstracts, two authors independently assessed 204 papers. Of those 204 papers, 159 publications were unrelated to AKI and therefore excluded from the study. Forty-five papers received a full-text review, and 23 were excluded based on the exclusion criteria. The flow diagram of the selection process is shown in Fig. 1. Finally, 22 studies including 16,199 COVID-19 patients met the predefined inclusion criteria and were used to determine the incidence of AKI in COVID-19 patients. Five of the 22 studies including 972 patients were used to determine the incidence of AKI in COVID-19 patients being treated with remdesivir.

Table 1 shows the characteristics of the studies in this systematic review. All studies in our systematic review reporting the incidence of AKI were retrospective cross-sectional studies, and most of them were of high quality (12/19). The RCTs included in our study were also of high quality.

**Table 1:** Characteristics of the studies included in the analysis of the incidence of AKI in hospitalized COVID-19 patients

Study	Year	Country	Design	Sample size	Age (median/ mean)	Male (%)	The diagnosis criteria of AKI	Department	Quality score
Yichun Cheng [6]	2020	China, Wuhan	Retrospective Cross-sectional study	701	63	52.4%	2012 KDIGO criteria Stage 1(*n* = 13) Stage 2 (*n* = 9) Stage 3(*n* = 14)	Hospitalized Patients	AHRQ 8
Weijie Guan [13]	2020	China, Wuhan	Retrospective Cross-sectional study	1099	47	58.1%	2012 KDIGO criteria	Hospitalized Patients	AHRQ 9
Chaolin Huang [14]	2020	China, Wuhan	Retrospective Cross-sectional study	41	49	73.0%	2012 KDIGO criteria CRRT 3(7%)	Hospitalized Patients	AHRQ 8
Shaobo Shi [15]	2020	China, Wuhan	Retrospective Cross-sectional study	416	64	49.7%	2012 KDIGO criteria CRRT 2(0.5%)	Hospitalized Patients	AHRQ 9
Luwen Wang [16]	2020	China, Wuhan	Retrospective Cross-sectional study	116	54	57.8%	2012 KDIGO criteria	Hospitalized Patients	AHRQ 6
Dawei Wang [17]	2020	China, Wuhan	Retrospective Cross-sectional study	138	56	54.3%	2012 KDIGO criteria CRRT 2(1.45%)	Hospitalized Patients	AHRQ 8
Fei Zhou [18]	2020	China, Wuhan	Retrospective Cross-sectional study	191	56	62.0%	2012 KDIGO criteria CRRT 10(5%)	Hospitalized Patients	AHRQ 8
Dawei Wang [19]	2020	China, Wuhan and Huanggang	Retrospective Cross-sectional study	107	51	53.3%	2012 KDIGO criteria	Hospitalized Patients	AHRQ 7
Tao Chen [20]	2020	China, Wuhan	Retrospective Cross-sectional study	274	62.0	62.4%	2012 KDIGO criteria CRRT 3(1%)	Hospitalized Patients	AHRQ 8
Xiaochen Li [21]	2020	China, Wuhan	Retrospective Cross-sectional study	548	60	50.9%	2012 KDIGO criteria CRRT 2(0.4%)	Hospitalized Patients	AHRQ 8
Xiaobo Yang [22]	2020	China, Wuhan	Retrospective Cross-sectional study	52	51.9	70%	2012 KDIGO criteria CRRT 9(17%)	ICU Patients	AHRQ 7
Yuan Yu [23]	2020	China, Wuhan	Retrospective Cross-sectional study	226	64	61.5%	2012 KDIGO criteria Stage 1 (*n* = 23); Stage2 (*n* = 12); Stage 3 (*n* = 22)	ICU Patients	AHRQ 7
KyungSoo Hong [24]	2020	Korea, Daegu	Retrospective Cross-sectional study	98	55.4	38.8%	2012 KDIGO criteria CRRT 3(3.1%)	Hospitalized Patients	AHRQ 6
Safiya Richardson [25]	2020	USA, New York	Retrospective Cross-sectional study	5700	63	60.3%	2012 KDIGO criteria CRRT 81(3.2%)	Hospitalized Patients	AHRQ 8
Jamie S. Hirsch [5]	2020	USA, New York	Retrospective Cross-sectional study	5449	64.0	60.9%	2012 KDIGO criteria CRRT 285(5.2%)	Hospitalized Patients	AHRQ 8
Jessica Ferguson [26]	2020	USA, California	Retrospective Cross-sectional study	72	60.4	52.8%	2012 KDIGO criteria	Hospitalized Patients	AHRQ 6
Matt Arentz [27]	2020	USA, Washington	Retrospective Cross-sectional study	21	79	52%	2012 KDIGO criteria	ICU Patients	AHRQ 8
J.H. Beigel [28]	2020	United States, Denmark, the United Kingdom,Greece, Germany, Korea, Mexico, Spain, Japan, and Singapore	RCT (Remdesivir)	1062	58.9	64.3%	2012 KDIGO criteria	Hospitalized Patients	Cochrane A
Spinello Antinori [29]	2020	Italy, Milan	Prospective, Cross-sectional study (Remdesivir)	35	63.0	74.3%	2012 KDIGO criteria	Hospitalized Patients	AHRQ 6
J. Grein [30]	2020	United States, Japan, Italy, Austria, France, Germany, Netherlands, Spain, and Canada	Prospective, Cross-sectional study (Remdesivir)	53	64	75%	2012 KDIGO criteria	Hospitalized Patients	AHRQ 8
Yeming Wang [31]	2020	China, Wuhan	RCT (Remdesivir)	236	66.0	56%	2012 KDIGO criteria	Hospitalized Patients	Cochrane A
Jason D. Goldman [32]	2020	United States, Italy, Spain, Germany, Hong Kong, Singapore, South Korea, and Taiwan	RCT (Remdesivir)	397	62	64%	2012 KDIGO criteria	Hospitalized Patients	Cochrane A

### Incidence of AKI in COVID-19 patients

**Fig. 2:**
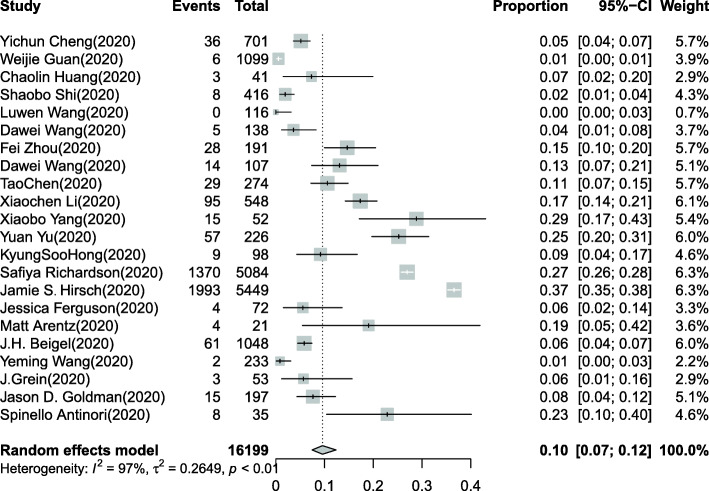
Forest plot of the incidence of AKI in COVID-19 patients. Overall, 16,199 COVID-19 patients in 22 studies were included*. I*^*2*^ > 50% indicated that heterogeneity existed among the studies. The random-effects model was used to pool the data. The pooled estimated incidence of AKI in all hospitalized COVID-19 patients was 10% (95% CI: 7–12%)

**Fig. 3:**
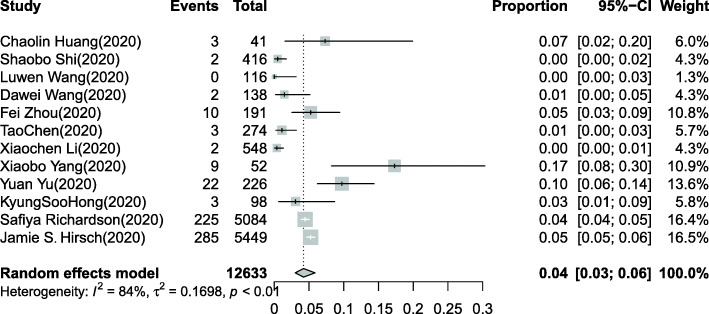
Forest plot of the proportion of COVID-19 patients who needed CRRT. A total of 12,633 COVID-19 patients in 12 studies were included*. I*^*2*^ > 50% indicated that heterogeneity existed among the studies. The random-effects model was used to pool the data. The pooled estimated proportion of hospitalized COVID-19 patients who needed CRRT was 4% (95% CI: 3–6%)

Overall, 16,199 COVID-19 patients were included in our systematic review [5, 6, 13–32]. The pooled estimated incidence of AKI in all hospitalized COVID-19 patients was 10% (95% CI: 7–12%, Fig. 2), and significant heterogeneity (I^2^ = 97%, chi-square = 0.26, *P* < 0.0001) was observed. Meanwhile, a total of 12,633 COVID-19 patients in 12 studies were included to investigate the need for CRRT [5, 14–18, 20–25]. A total of 566 patients (15.6%) needed CRRT among 3612 COVID-19 patients with AKI. The pooled estimated proportion of COVID-19 patients who needed CRRT was 4% (95% CI: 3–6%, Fig. 3).

### Incidence of AKI in different subgroups of COVID-19 patients

Subgroup analyses were performed according to ethnicity, age and disease severity (Supplementary Fig. 1, 2, 3). The pooled estimated AKI incidences in the Asian subgroup and non-Asian subgroup were 7% (95% CI: 4–11%) and 15% (95% CI: 11–20%), respectively (Supplementary Fig. 1). At the same time, the incidences of AKI in the subgroup with a median/mean age greater than 60 years and the subgroup with a median/mean age less than 60 years were 12% (95% CI: 9–16%) and 6% (95% CI: 3–12%), respectively (Supplementary Fig. 2). In the subgroup of hospitalized patients, the incidence of AKI was 8% (95% CI: 6–11%), but it was 26% (95% CI: 21–31%) in ICU patients (Supplementary Fig. 3). There was still significant heterogeneity in most of the subgroups in our subgroup analysis.

### Incidence of AKI in the subgroup of COVID-19 patients being treated with remdesivir

**Fig. 4:**
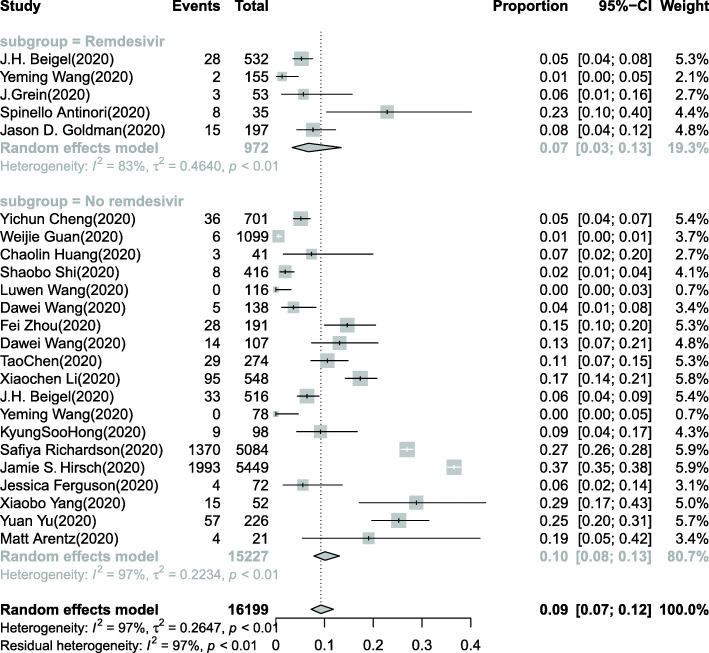
Forest plot of the incidence of AKI in the remdesivir and no remdesivir subgroups of COVID-19 patients. A total of 972 COVID-19 patients in 5 studies were included in the remdesivir subgroup, and 15,227 patients were included in the no remdesivir subgroup*.* The pooled estimated incidence of AKI in COVID-19 patients being treated with remdesivir was 7% (95% CI: 3–13%). In the no remdesivir subgroup of COVID-19 patients, the incidence of AKI was 10% (95% CI: 8–13%)

A total of 5 studies with 972 COVID-19 patients investigated the incidence of AKI in hospitalized COVID-19 patients being treated with remdesivir [28–32]. The pooled estimated AKI incidence in hospitalized COVID-19 patients being treated with remdesivir was 7% (95% CI: 3–13%) (Fig. 4). In the subgroup of COVID-19 patients not treated with remdesivir, the incidence of AKI was 10% (95% CI: 8–13%).

### Sensitivity analysis and publication bias

In the sensitivity analysis, we used the leave-one-out method (Supplementary Figs. 4 and 5) and found similar results to those in our main study. Peter’s test was performed to evaluate publication bias (Table 2), and no significant difference was detected in the incidence of AKI in COVID-19 patients.

**Table 2:** Results of the systematic review of the incidence of AKI and the proportion of patients who needed CRRT among all COVID-19 patients

	Study No.	COVID-19 patients No.	Proportion/OR (95%CI)	Study heterogeneity			
				Chi-square test	df	I^2^	Peter’s test (*P* value)
The incidence of AKI in COVID-19 patients	22	16,199	0.10(0.07–0.12)	0.26	21	97%	0.18
The incidence of CRRT in COVID-19 patients	12	12,633	0.04(0.03–0.06)	0.17	11	84%	0.24

## Discussion

In this systematic review, the results from 22 retrospective cross-sectional studies including 16,199 patients hospitalized with COVID-19 from 1 January 2020 to 1 June 2020 demonstrated that AKI was not rare in COVID-19 patients. The incidence of AKI might be associated with age, disease severity and ethnicity, according to our subgroup analyses.

COVID-19 is primarily a respiratory disease, but other organs, including the kidneys, are often involved. SARS-CoV-2 enters cells via the angiotensin-converting enzyme 2 (ACE2) receptor and is highly homologous to SARS-CoV [33]. High ACE2 expression in proximal tubular epithelial cells may make the kidneys a potential target, leading to kidney injury [34]. Renal abnormalities, such as proteinuria, haematuria, and AKI, occur in patients with COVID-19 [35]. AKI is characterized by a rapid increase in serum creatinine, a decrease in urine output, or both [36]. The current widely used AKI definition was developed by the Kidney Disease Improving Global Outcomes (KDIGO) group in 2012 [37]. The most common causes of AKI are septic shock, major surgery, cardiogenic shock, drug toxicity and hypovolemia [38]. The cause of AKI in COVID-19 patients is likely to be multifactorial, including a direct attack by SARS-CoV-2 (COVID-19-associated acute kidney injury: consensus report of the 25th Acute Disease Quality Initiative (ADQI) Workgroup) or haemodynamic instability, microcirculatory dysfunction, tubular cell injury, renal congestion, microvascular thrombi and endothelial dysfunction [39], which are commonly found in critically ill patients. Pathological reports from autopsies of patients with COVID-19 with renal failure revealed that the kidneys contained viral particles within both the tubular epithelium and the podocytes that were visible with electron microscopy [40], varying degrees of acute tubular necrosis (ATN), diffuse proximal tubule injury with the loss of the brush border, nonisometric vacuolar degeneration, haemosiderin granules and pigmented casts [40, 41].

We found that the incidence of AKI in COVID-19 patients was 10%. A similar AKI incidence in COVID-19 patients (10.8%) was also reported in another study [34]. The diversity of patients included in our systematic review resulted in heterogeneity. According to the subgroup analysis, the estimated AKI incidence in patients with an average age greater than 60 years old was 12%, while that in patients with an average age less than 60 years old was 6%. Many reports on COVID-19 have highlighted age-related differences in health outcomes, and the mortality due to COVID-19 is particularly high among older patients [42, 43]. Age is also an important risk factor for AKI [44]. The pooled estimated AKI incidence in the Asian subgroup was 7%. However, in the non-Asian subgroup, it was 15%. African ancestry is also a risk factor for AKI [45]. In a large cohort study of hospitalized COVID-19 patients, 76.9% of the patients who were hospitalized with COVID-19 and 70.6% of those who died were Black, whereas the Black population only accounted for 31% of the total population [46]. There might be a difference between the criteria for hospital admission in Asian and non-Asian COVID-19 patients. A European study showed that 190/1457 (13%) COVID-19 patients were diagnosed with AKI on arrival [47]. The incidence of AKI in ICU patients with COVID-19 is particularly high, ranging from 8 to 62% [14, 17, 22–24, 26, 27]. In our subgroup analysis, we found that the incidence of AKI was 26% in ICU patients. Critically ill patients hospitalized with COVID-19 who stayed in the ICU were more likely to develop AKI [5]. Lin L proved that disease severity was associated with the incidence of AKI in COVID-19 patients [34].

The proportion of COVID-19 patients who needed CRRT was 4%, according to our investigation. CRRT has been administered to many sepsis patients complicated with AKI [48]. Growing evidence suggests that patients with severe COVID-19 may develop cytokine storm syndrome [49, 50]. CRRT can remove inflammatory factors, thus blocking cytokine storm syndrome and ultimately reducing the damage inflicted on multiple organs [51]. However, the timing of the initiation of CRRT in patients with severe COVID-19 remains controversial [49]. Additional research is needed to determine whether the early initiation of CRRT could improve the prognosis of COVID-19 patients with AKI.

The initiation of treatment with antiviral drugs is a common cause of drug-induced AKI [52, 53]. As shown in Fig. 4, the incidence of AKI in hospitalized COVID-19 patients being treated with remdesivir was 7%. In clinical studies of remdesivir, AKI was the most frequent adverse event leading to drug discontinuation [29, 31]. Antiviral drugs cause AKI through many mechanisms, including direct renal tubular toxicity, allergic interstitial nephritis (AIN), and crystal nephropathy [54, 55]. However, in animal models, remdesivir was effective against MERS-CoV and did not cause any side effects, such as AKI [56]. According to a recently published multicentre matched cohort study of remdesivir, remdesivir was not significantly associated with an increased incidence of AKI in COVID-19 patients, even in patients who had a baseline eCrCl< 30 mL/min [57]. In our study, we also did not observe remdesivir-associated AKI in COVID-19 patients. More RCTs should be performed on this topic in the future.

### Limitations

Our systematic review had some limitations. First, most of the studies included were retrospective cross-sectional studies, although the majority of them (65%) were of high quality. Second, the systematic review was performed using studies with single groups, leading to greater heterogeneity. There was statistically significant heterogeneity in the systematic review of the incidence of AKI in COVID-19 patients. The diversity of the included studies, which involved different disease stages or activities, ages, ethnicities and sexes, might also be associated with the heterogeneity. Although we performed subgroup analyses, the results still had significant heterogeneity. As COVID-19 is a new and unknown infectious disease, our review could only summarize the studies that have already been published on this topic. The potential bias in the reported COVID-19 patients means that they may not represent all of the patients hospitalized with COVID-19 worldwide. Third, there were few original studies (*n* < 10) that could be included in the systematic review of the incidence of AKI in hospitalized COVID-19 patients being treated with remdesivir. Finally, since investigations of COVID-19 are ongoing, additional clinical data are expected to be published.

## Conclusion

According to our study, AKI is common in hospitalized COVID-19 patients. The incidence of AKI could be associated with age, disease severity and ethnicity. Remdesivir probably does not induce AKI in COVID-19 patients. Our systematic review demonstrated the clinical characteristics of AKI in COVID-19 patients, providing evidence that AKI might be closely associated with SARS-CoV-2 infection, which should be assessed in future studies.

## Supplementary Information

**Additional file 1: Figure S1.** Forest plot of the incidence of AKI in the Asian and non-Asian subgroups of COVID-19 patients.

**Additional file 2: Figure S2.** Forest plot of the incidence of AKI in the median/mean age more than 60 years and less than 60 years subgroups of COVID-19 patients.

**Additional file 3: Figure S3.** Forest plot of the incidence of AKI in the ICU and hospitalized subgroups of COVID-19 patients.

**Additional file 4: Figure S4.** Sensitivity analysis for the incidence of AKI in COVID-19 patients.

**Additional file 5: Figure S5.** Sensitivity analysis for the proportion of COVID-19 patients who needed CRRT.

## Data Availability

The datasets used and/or analysed during the current study are available from the corresponding author upon reasonable request.

## Abbreviations

AKI: Acute kidney injury
COVID-19: Coronavirus disease 2019
CRRT: Continuous renal replacement therapy
ICU: Intensive care unit
SARS-CoV-2: Severe acute respiratory syndrome coronavirus 2
EV: Ebola virus
RCTs: Randomized controlled trials
