# Supplementary figures and images for: Systematic review and subgroup analysis of the incidence of acute kidney injury (AKI) in patients with COVID-19
Source: BMC Nephrol. 2021 Feb 5;22:52. doi: 10.1186/s12882-021-02244-x (PMC7863041; doi:10.1186/s12882-021-02244-x)

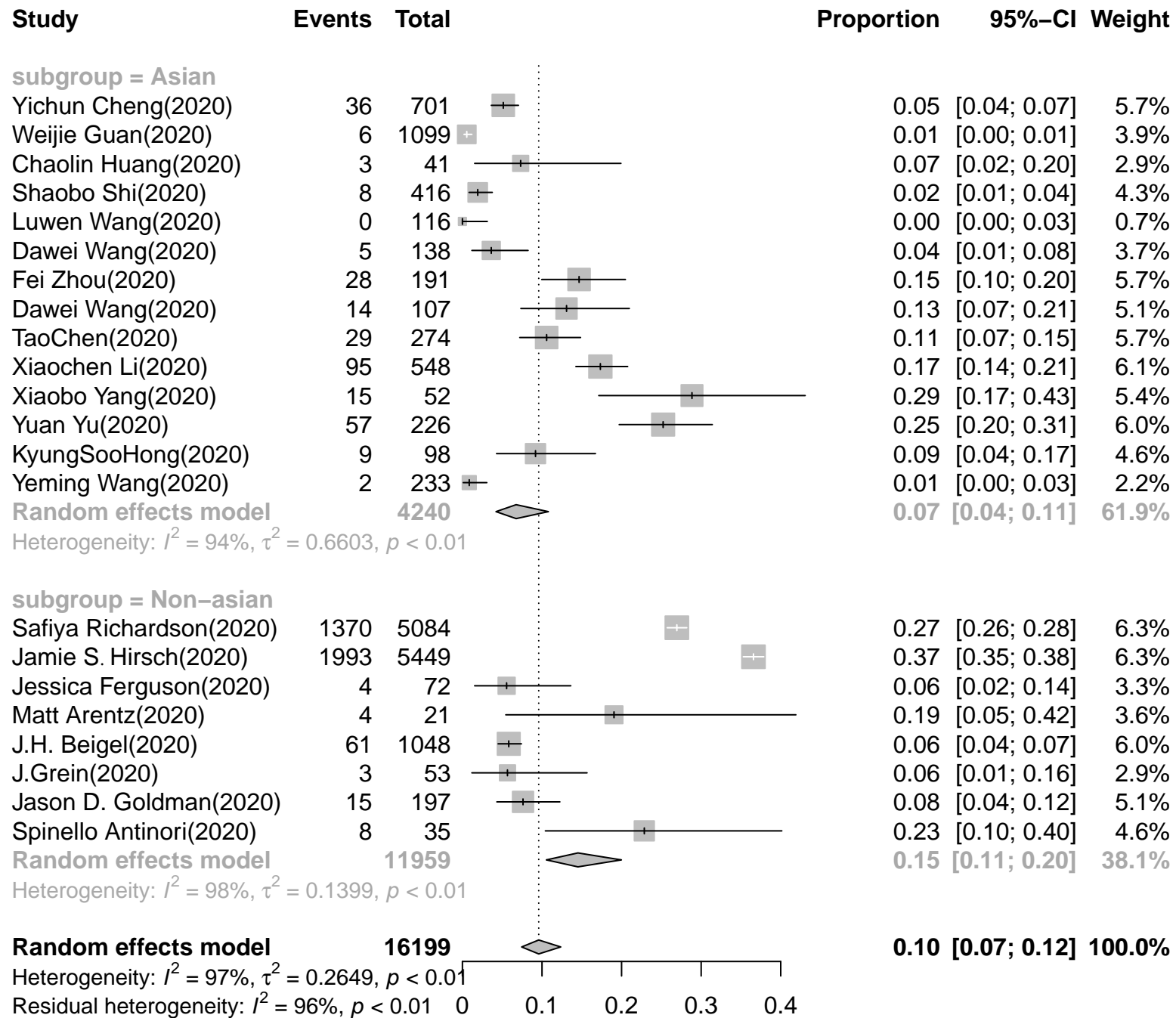

Supplement: Supplementary file 1 — Additional file 1: Figure S1. Forest plot of the incidence of AKI in the Asian and non-Asian subgroups of COVID-19 patients. [file 12882_2021_2244_MOESM1_ESM.pdf]

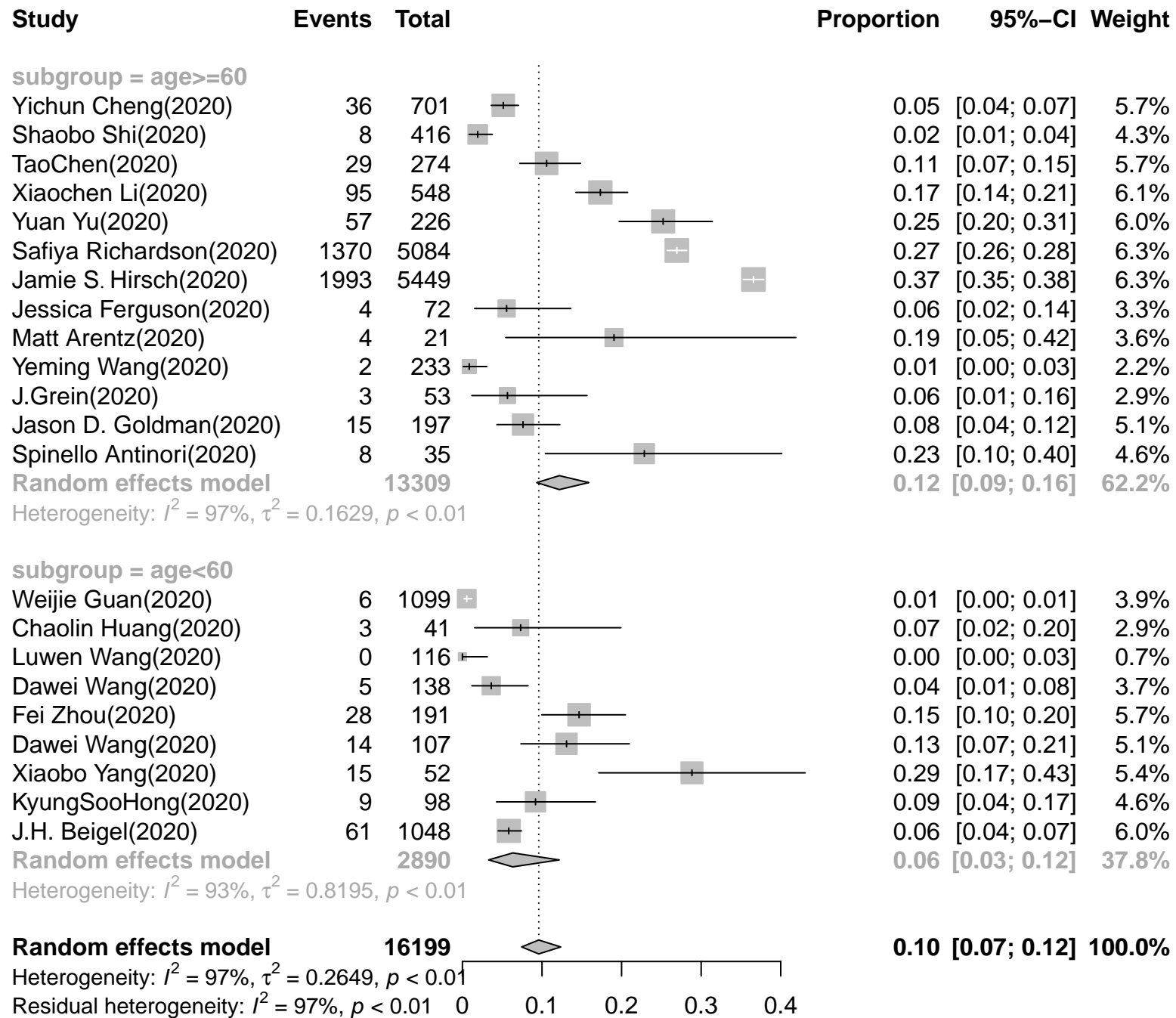

Supplement: Supplementary file 2 — Additional file 2: Figure S2. Forest plot of the incidence of AKI in the median/mean age more than 60 years and less than 60 years subgroups of COVID-19 patients. [file 12882_2021_2244_MOESM2_ESM.pdf]

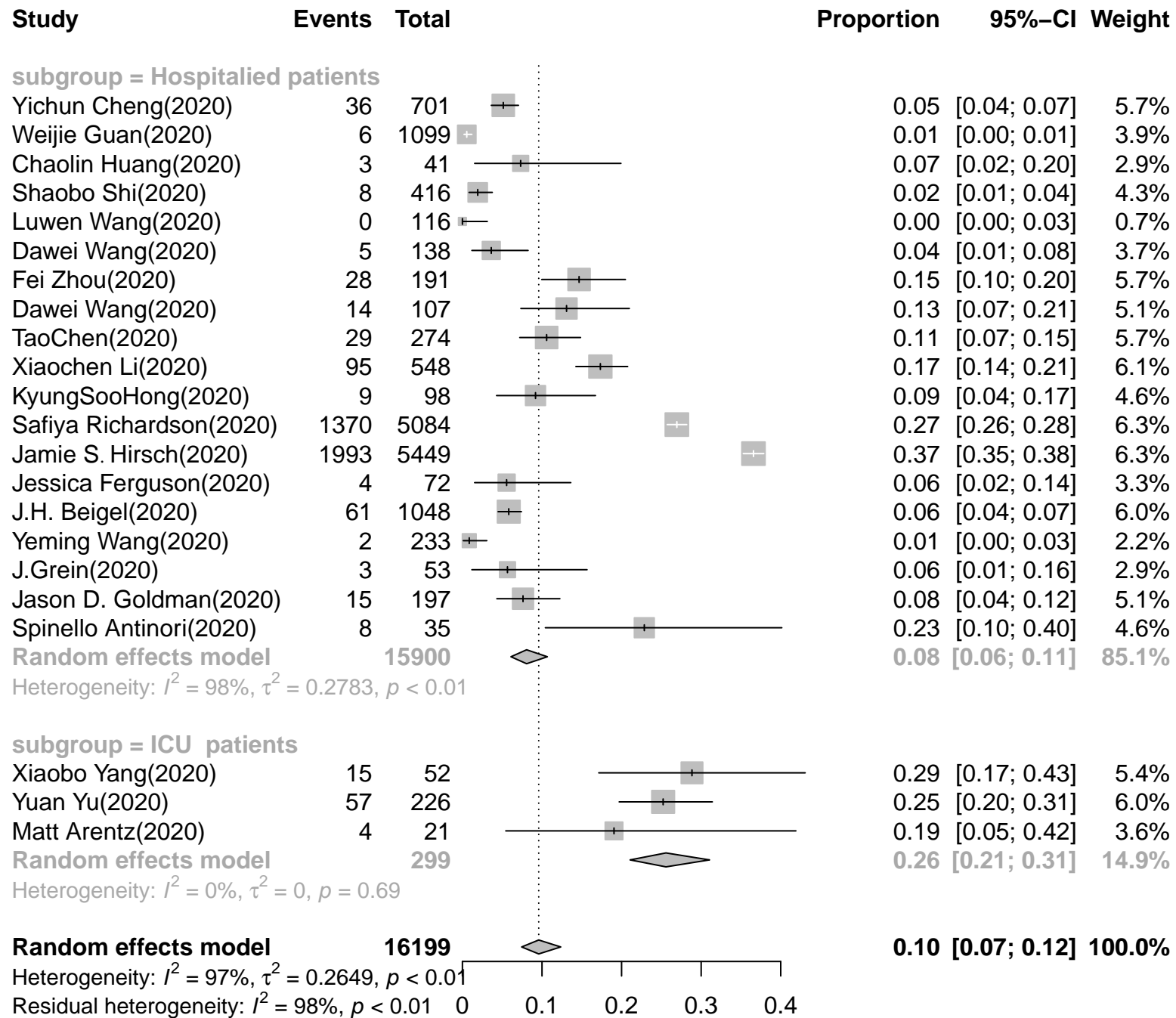

Supplement: Supplementary file 3 — Additional file 3: Figure S3. Forest plot of the incidence of AKI in the ICU and hospitalized subgroups of COVID-19 patients. [file 12882_2021_2244_MOESM3_ESM.pdf]

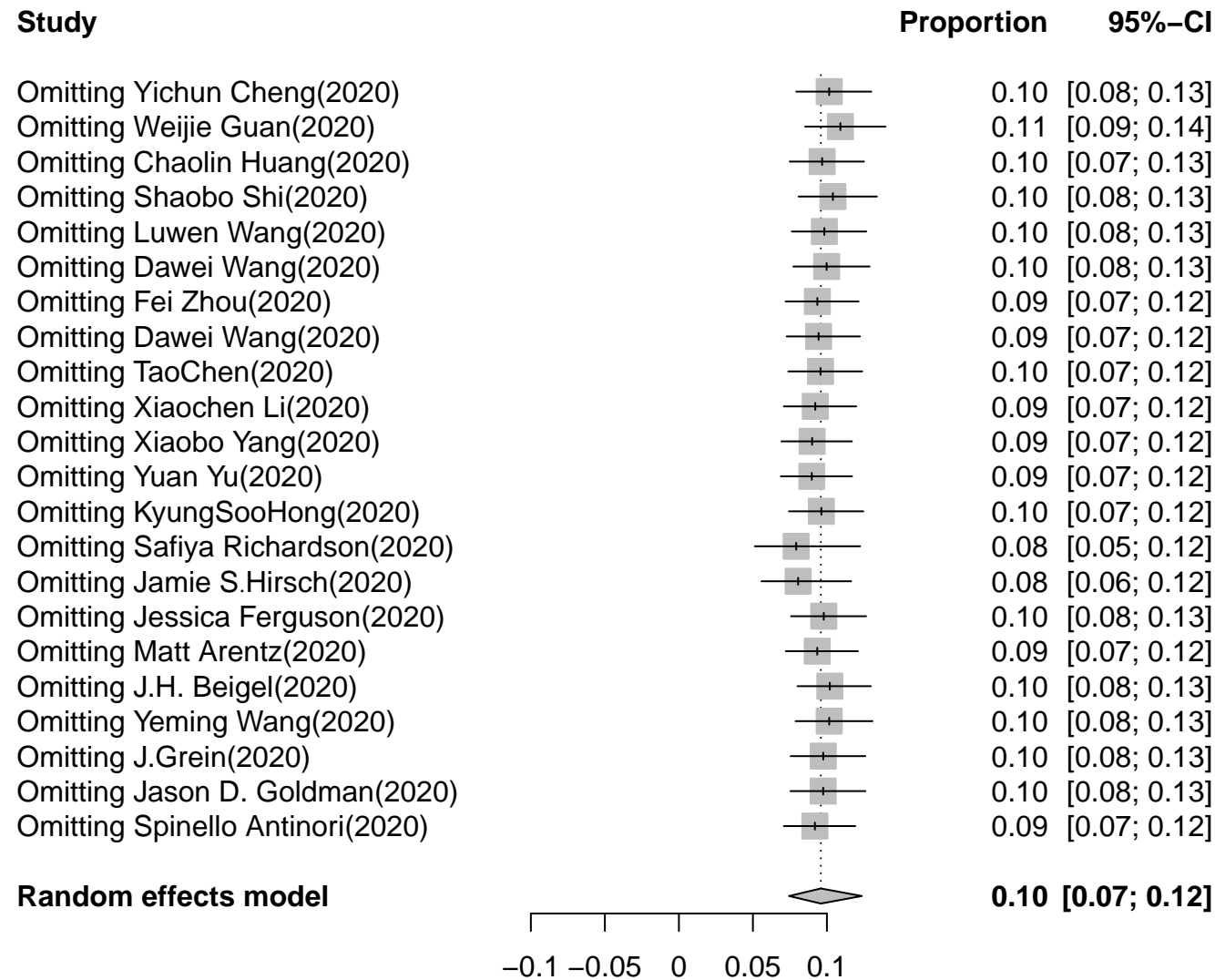

Supplement: Supplementary file 4 — Additional file 4: Figure S4. Sensitivity analysis for the incidence of AKI in COVID-19 patients. [file 12882_2021_2244_MOESM4_ESM.pdf]

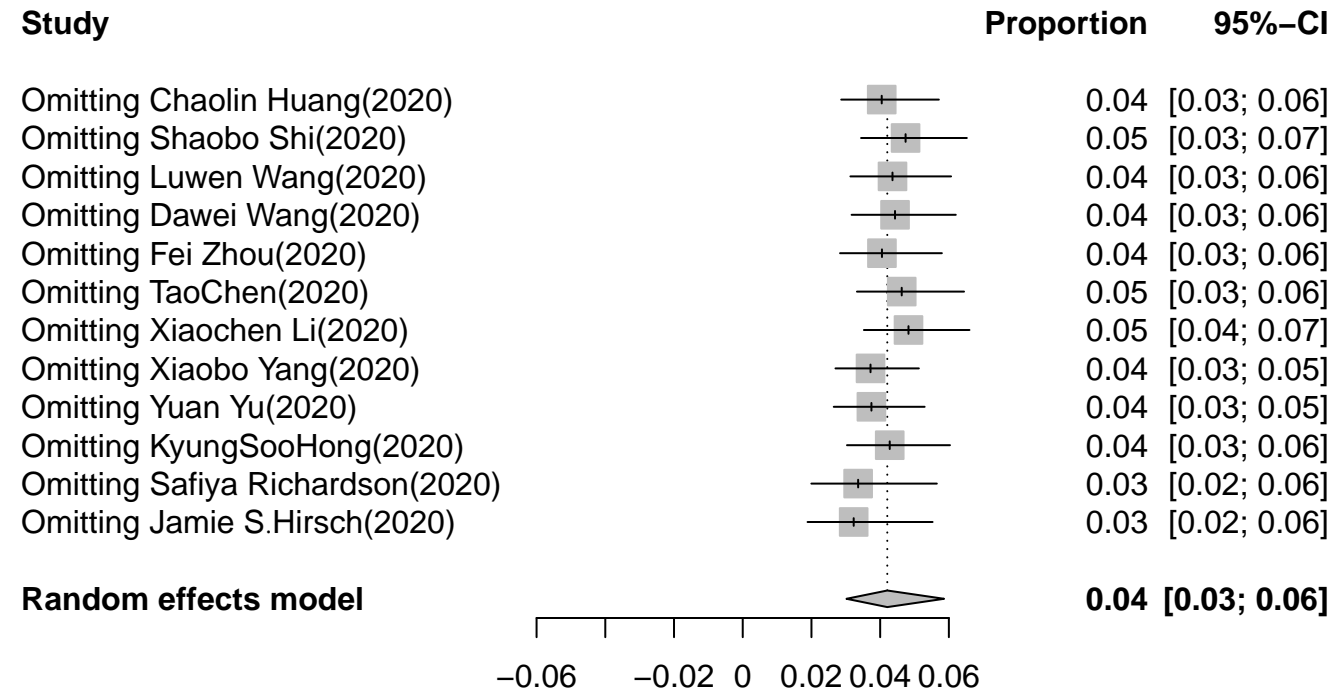

Supplement: Supplementary file 5 — Additional file 5: Figure S5. Sensitivity analysis for the proportion of COVID-19 patients who needed CRRT. [file 12882_2021_2244_MOESM5_ESM.pdf]
